# Respiratory Diaphragm Motion-Based Asynchronization and Limitation Evaluation on Chronic Obstructive Pulmonary Disease

**DOI:** 10.3390/diagnostics13203261

**Published:** 2023-10-20

**Authors:** Xingyu Zhou, Chen Ye, Yuma Iwao, Takayuki Okamoto, Naoko Kawata, Ayako Shimada, Hideaki Haneishi

**Affiliations:** 1 Graduate School of Science and Engineering, Chiba University, Chiba 263-8522, Japan; x_zhou@chiba-u.jp (X.Z.);; 2 School of Communications and Information Engineering, Nanjing University of Posts and Telecommunications, Nanjing 210003, China; 3 Center for Frontier Medical Engineering, Chiba University, Chiba 263-8522, Japan; 4 National Institutes for Quantum and Radiological Science and Technology, Chiba 263-0024, Japan; 5 Department of Respirology, Graduate School of Medicine, Chiba University, Chiba 260-0856, Japan; romanholiday52@yahoo.co.jp; 6 Department of Respirology, Shin-Yurigaoka General Hospital, Kawasaki 215-0026, Japan

**Keywords:** chronic obstructive pulmonary disease (COPD), respiratory diaphragm motion, magnetic resonance imaging (MRI), field segmentation, respiration cycle

## Abstract

**Background:** Chronic obstructive pulmonary disease (COPD) typically causes airflow blockage and breathing difficulties, which may result in the abnormal morphology and motion of the lungs or diaphragm. **Purpose:** This study aims to quantitatively evaluate respiratory diaphragm motion using a thoracic sagittal magnetic resonance imaging (MRI) series, including motion asynchronization and limitations. **Method:** First, the diaphragm profile is extracted using a deep-learning-based field segmentation approach. Next, by measuring the motion waveforms of each position in the extracted diaphragm profile, obvious differences in the independent respiration cycles, such as the period and peak amplitude, are verified. Finally, focusing on multiple breathing cycles, the similarity and amplitude of the motion waveforms are evaluated using the normalized correlation coefficient (NCC) and absolute amplitude. **Results and Contributions:** Compared with normal subjects, patients with severe COPD tend to have lower NCC and absolute amplitude values, suggesting motion asynchronization and limitation of their diaphragms. Our proposed diaphragmatic motion evaluation method may assist in the diagnosis and therapeutic planning of COPD.

## 1. Introduction

Chronic obstructive pulmonary disease (COPD) is an obstructive lung disease that usually occurs in smokers or people who are often exposed to toxic substances [1,2,3]. In particular, COPD-related lung function disorders may result in difficulties in the patient’s daily activities, such as walking and climbing stairs [4,5,6]. In normal subjects, respiratory activity usually brings about a synchronized up-down movement for each. In contrast, owing to airflow limitations and diaphragmatic elasticity damage, the diaphragmatic motion of patients with COPD may be asynchronized and limited, which are respectively represented by the different, even opposite motion directions at different positions of the diaphragm, e.g., at anterior and posterior positions, and small motion amplitude.

Pulmonary function tests (PFTs) are common lung diagnostic methods used in clinical practice [7,8,9]. PFTs diagnose lung status by measuring air-volume-based indices, such as forced vital capacity (FVC) and forced expiratory volume in 1 s (FEV1) [10,11]. However, PFTs cannot evaluate the morphology or motion of the lungs or diaphragm. Numerous tomography imaging techniques that can provide structural lung information have been developed and applied in medicine and healthcare, including computed tomography (CT) and magnetic resonance imaging (MRI) [12,13]. Compared to CT with radiation, MRI can continuously acquire high-speed image data [14]. Using sequential MRI, the motion characteristics of the lungs and diaphragm can be observed and analyzed.

In the past decades, numerous studies on tomography-imaging-based motion assessments of the respiratory lung or diaphragm motion have been performed [15,16,17,18,19]. Chun et al. [15] used a chest X-ray to evaluate diaphragm motion before and after pulmonary rehabilitation for patients with COPD by measuring the area of motion of the diaphragm between maximum inspiration and expiration, which can help verify the effects of rehabilitation. Hida et al. [16] quantitatively tracked the displacement and speed of a specified position at the diaphragm to analyze diaphragm motion in COPD patients using dynamic chest radiography. Yamashiro et al. [17] used four-dimensional dynamic-ventilation CT to calculate the mean lung densities (MLDs), based on the X-ray attenuation of pulmonary tissue [20], around several specified positions of the pulmonary lobes, whose variation corresponded to the respiration phase. The asynchronized motion of the pulmonary lobes of COPD patients was evaluated by observing the MLD. Suga et al. [18] used dynamic breathing MRI (BMRI) data to measure the height of the lung and the thickness of the chest wall during approximately two respiration cycles to quantitatively evaluate the impaired respiratory mechanics of patients. Sato et al. [19] first extracted the lung area using the clustering approach, termed *K*-means [21], and the asynchronized motion of the diaphragm was assessed by comparing the displacement of three equispaced positions on the diaphragm. In addition, the relationships between the extracted lung area and other clinical indices, such as FVC and FEV1, were investigated.

Conventional evaluation methods for lung and diaphragm motion are limited in two ways. First, most conventional methods focus only on motion during very few respiration cycles, namely, one or two cycles [15,16,17,18]. However, obvious waveform differences exist among the respiration cycles for each subject, including the period and the peak amplitude. Second, some conventional methods only investigate the motions of relatively few positions in the lung or on the diaphragm, which may ignore some motion characteristics for some positions [16,18,19]. These two limitations with conventional methods motivated us to propose a novel diaphragm motion evaluation method that considers continuous respiration activity with multiple cycles and all positions in the diaphragm profile.

This study aims to investigate the differences in respiratory diaphragm motion between normal subjects and COPD patients, especially for the motion asynchronization and limitation of the diaphragm, assisting in the diagnosis and therapy of COPD and even the corresponding rehabilitation of patients. Considering the limitations of conventional methods, in this study, we develop a diaphragm motion evaluation method based on multiple respiration cycles, consisting of three main approaches. First, we extract the diaphragm profiles from the thoracic sagittal MRI series using a deep-learning-based field segmentation technique [22,23] named U-net [24,25,26]. The temporal motion waveforms generated from the vertical changes of each position in the extracted diaphragm profile can be naturally obtained. Second, the period and peak amplitude differences among multiple respiration cycles are quantitatively verified for all subjects. Finally, two metrics, the normalized correlation coefficient (NCC) [27,28] and the absolute amplitude, are used to evaluate the similarity and magnitude of the obtained motion waveforms, reflecting the asynchronization and limitation of the diaphragm motion.

The two main contributions of this study are summarized as follows.

1. Using a thoracic MRI series for a relatively long time, a novel multiple-respiration-cycle-based method is developed to reliably evaluate diaphragm motion.
2. To investigate motion asynchronization and the limitation of diaphragmatic motion of patients with COPD, two new evaluation metrics are utilized: NCC and absolute amplitude.

The remainder of this paper is organized as follows. Section 2 describes the materials and proposed diaphragm motion evaluation method; Section 3 presents the experiments and results; Section 4 presents a discussion; and finally, Section 5 concludes the study with future possible research directions.

## 2. Materials and Methods

### 2.1. Materials

#### 2.1.1. Participants

This retrospective study enrolled 20 patients who were diagnosed with COPD and underwent chest dynamic MRI at Chiba University Hospital, Japan, from April 2011 to September 2018. Most of the participants in the present study had been investigated in the previous study with a different research objective [29]. The subjects were required to meet all of the following inclusion criteria: (a) ≥40 years old; (b) smoking history ≥10 pack-years; (c) COPD diagnosed or suspected to have COPD based on subjective symptoms/other findings/pulmonary function tests/imaging findings. The exclusion subjects are subject to the following criteria: (a) Obvious respiratory diseases other than COPD; (b) severe heart failure; and (c) deemed unsuitable for inclusion by investigators for any other reason. All patients had a history of smoking. COPD was diagnosed according to the criteria of the Global Initiative for Chronic Obstructive Lung Disease (GOLD) [30,31]. The patients underwent a PFT and MDCT imaging within 4 months of undergoing MRI. Two subjects were excluded, one with a sagittal plane image deficiency and one with an imaging issue resulting in shadows around the sagittal diaphragm images. The normal control participants consisted of 10 healthy subjects who underwent PFT and MRI. Finally, ten normal subjects and eighteen patients diagnosed with COPD were enrolled in the study.

#### 2.1.2. MRI

A 1.5-T Ingenia CX/Achieva dStream Release 5MR system (Phillips Medical Systems, Amsterdam, The Netherlands) was used to capture dynamic thoracic MR images from the sagittal plane. The parameters of the MRI system are summarized in Table 1. A balanced fast-field echo sequence was set (repetition time, 1.84 ms; echo time, 0.71 ms; flip angle, 45°). The size of the captured MR image was 256×256 pixels, corresponding to a field of view of 384×384 mm, with a slice thickness of 13.5 mm. At a frame rate of 8.33 f/s, at least $M_{ori}=1200$ MR images were acquired for over 2 min for each subject.

### 2.2. Method

**Figure 1:**
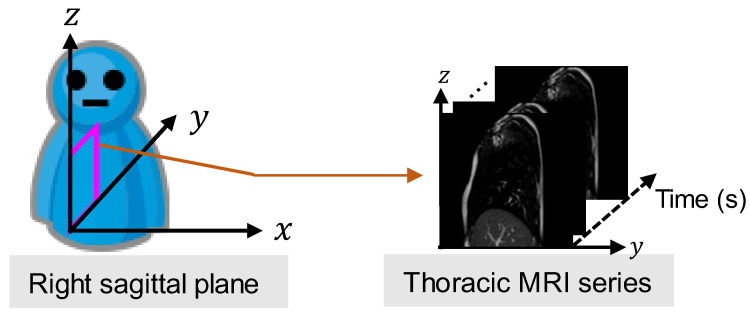
Magnetic resonance imaging (MRI) series captured from a sagittal plane of the right lung.

**Figure 2:**
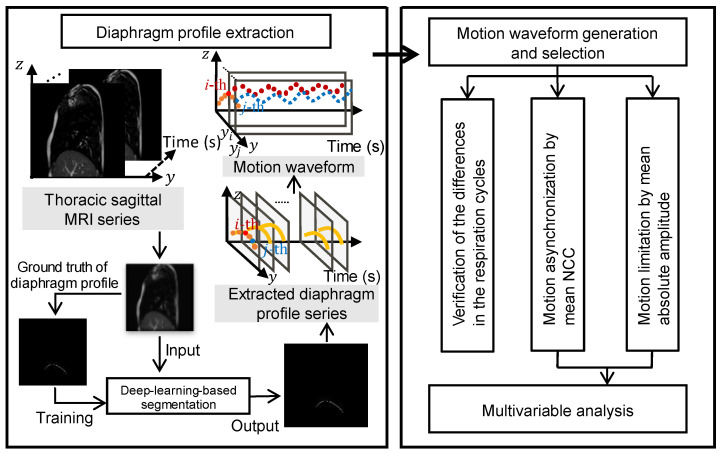
Flowchart of the proposed diaphragm motion evaluation method.

The coordinate system of the MR image is shown in Figure 1. The X-, Y-, and Z-coordinates represent the right-left (RL), anterior-posterior (AP), and superior-inferior (SI) directions, respectively. A flowchart of the proposed diaphragm motion evaluation method is depicted in Figure 2. The method consisted of four main steps:

1. As a pre-processing step, the trained field segmentation model of U-net [24] is used to extract the diaphragm profile series from the captured thoracic sagittal MRI series, and the time-varying vertical motion waveforms of the height for each position on the diaphragm profile were naturally generated.
2. The differences in the period and peak amplitude of the respiration cycles are verified for each subject by the motion waveforms, which motivates us to measure and evaluate diaphragm motion with multiple cycles.
3. Considering multiple respiration cycles, the asynchronization of diaphragm motion is evaluated by calculating the mean NCC value among all motion waveforms.
4. In addition, considering multiple respiration cycles, the limitation of diaphragm motion is evaluated by calculating the mean absolute amplitude of the partial motion waveforms.

#### 2.2.1. Diaphragm Profile Extraction

Because the lung or diaphragm on the right side typically has a larger volume or area than the lung or diaphragm on the left side, we chose the right diaphragm for profile extraction to better analyze diaphragm motion. Specifically, we employed 8400 thoracic sagittal MR images and their corresponding ground truths for diaphragm profiles from two normal subjects and five COPD patients to train the U-net segmentation model, as illustrated in Figure 2. The trained U-net model was used to extract diaphragm profiles from the prediction dataset, and a dice accuracy value of over 97% was confirmed. The accurate motion waveforms generated were helpful for the following diaphragm motion analyses: i.e., respiration cycle difference verification, motion asynchronization, and limitation evaluations.

We define the binary flag of the extracted diaphragm profile, $f\left(y,t\right)$, as follows:

(1) $$f\left(y,t\right)=\left\{\begin{array}{cc} 1, & if\;within\;the\;diaphragm\;field\\ 0, & otherwise \end{array}\right.,$$

Correspondingly, the position of the Z-axis of the extracted diaphragm profile corresponding to an arbitrary position $\left(y,t\right)$, consisting of the position of the Y-axis and moment, is defined as:

(2) $$z_{dia}(y,t)=\left\{\begin{array}{cc} z(y,t), & if\;f\left(y,t\right)=1\\ none, & otherwise \end{array}\right.,$$

The number of pixels corresponding to the extracted diaphragm profile, that is, the diaphragm profile length, at the m-th moment, is given by:

(3) $$N_{ori}(t_{m})=\sum_{i}f\left(y_{i},t_{m}\right),$$

#### 2.2.2. Respiration Cycle Difference Verification

The differences in the respiration cycle are represented by the motion waveforms of a position in the diaphragm profile, but these have been neglected in previous studies [15,16,17,18]. In a respiratory cycle, the period and the peak amplitude are generally deemed to be two key indices, and neglecting the difference in the period or peak amplitude may result in an inaccurate motion assessment of the lung or diaphragm. Therefore, we focus on the 2 min motion waveforms with multiple respiration cycles in the following diaphragm motion evaluation. To further explore the motion waveform differences quantitatively, statistics are employed to evaluate the differences in the motion period and motion peak amplitude, as presented in Section 3. Note that the mentioned peak amplitude is different from the absolute amplitude for evaluating motion limitation in Section 2.2.4.

#### 2.2.3. Motion Asynchronization Evaluation

For each position within the common range of all diaphragm profiles, the motion waveforms can be generated along the time (frame) axis. The length of the waveform equals the number of frames, for example, $M_{ori}=1200$, and the number of waveforms equals the length of the common range of the diaphragm profile in the view of the Y-axis, e.g., $N_{ori}$. However, in some cases, though the length of waveforms is close to $M_{ori}$, the common range of each diaphragm profile is overly short with a small common length $N_{ori}$, which may affect a comprehensive evaluation of diaphragm motion. Given that, we moderately extend the short common range by discarding some frames with relatively short diaphragm profiles.

**Figure 3:**
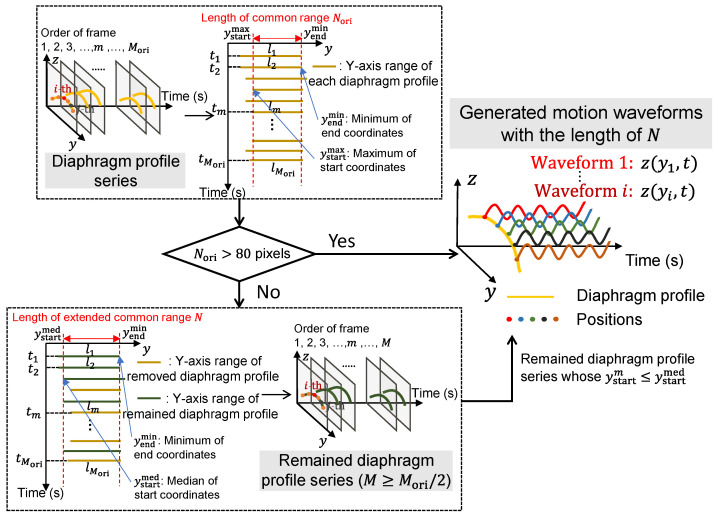
The selection mechanism of diaphragm profiles for the generated motion waveforms.

The selection mechanism for the diaphragm profiles for the generated motion waveforms is illustrated in Figure 3. Based on empirical observations, a common length of more than 80 pixels is considered sufficient for generating enough motion waveforms. When $N_{ori}$ is greater than 80 pixels, the $N_{ori}$ motion waveforms corresponding to $N_{ori}$ positions on the common diaphragm profile can be generated naturally with $M_{ori}=1200$ frames. In contrast, when $N_{ori}$ is not more than 80 pixels, the common range of the diaphragm profile will be extended by discarding some frames. For all the diaphragm profiles, the end of the common range $y_{end}^{min}$ calculated by the minimum of all the end coordinates (i.e., $y_{end}^{m}$) is unchanged, and the median of all the start coordinates (i.e., $y_{start}^{m}$) $y_{start}^{med}$ is a new start of the common range by replacing $y_{start}^{max}$, with the length of the extended common range $N=y_{end}^{min}-y_{start}^{med}$ and $M\geq M_{ori}/2$ diaphragm profiles. Subsequently, M remained diaphragm profiles with N-length are used for the following motion waveform generation.

Based on Equation (2), the temporal average of the Z-axis positions corresponding to the i-th position on the Y-axis can be calculated as follows:

(4) $$\bar{z}\left(y_{i}\right)=\frac{1}{M}\sum_{m}z_{dia}(y_{i},t_{m}),$$

where M denotes the number of diaphragm profile frames after removing frames with a short diaphragm profile length, which is equal to the number of frames selected. In addition, we defined the centralized positions of the Z-axis between the time-varying positions of $z(y_{i},t_{m})$ and $\bar{z}\left(y_{i}\right)$ as

(5) $$\hat{z}\left(y_{i},t_{m}\right)≔z\left(y_{i},t_{m}\right)-\bar{z}\left(y_{i}\right),m=1,2,\cdots ,M,$$

To investigate the asynchronization of the diaphragm motion of patients with COPD, the common metric, NCC [27], is adopted to calculate the similarity between each of the two motion waveforms of a position in the diaphragm profile. The NCC value is defined as:

(6) $$R_{ij}=\frac{\sum_{m=1}^{M}\hat{z}\left(y_{i},t_{m}\right)\hat{z}(y_{j},t_{m})}{\sqrt{\sum_{m=1}^{M}{\hat{z}(y_{i},t_{m})}^{2}}\sqrt{\sum_{m=1}^{M}{\hat{z}(y_{j},t_{m})}^{2}}},$$

where i and j denote two independent indices for the positions in the diaphragm profile.

**Figure 4:**
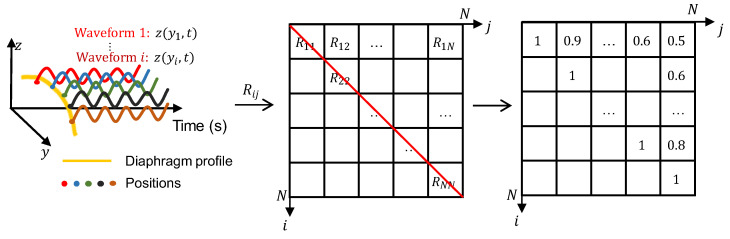
Construction of a normalized correlation coefficient (NCC) map for a subject and examples of NCC map.

To visualize the waveform similarity between two different positions, an NCC map is constructed, as shown in Figure 4. Corresponding to each position indexed by the pair of $\left[i,j\right]$, the NCCs calculated between each of the two motion waveforms are filled into each grid of the NCC map. It is easy to understand that the NCCs are equal to 1 along the diagonal, where indices i and j represent the same position, and those at the bottom left occupying half of the map are omitted owing to symmetry. In the NCC map, grids with large NCC values, such as 0.8 and 0.9, usually correspond to the positions of the diaphragm profile with high similarities. In contrast, grids with small NCC values, such as 0.5 and 0.6, usually correspond to the positions of the diaphragm profile with motion asynchronization.

Finally, all NCCs at the top right, except those along the diagonal, are used to calculate the mean NCC value, which will be used in the following multivariate analysis. The mean NCC value was calculated using $\bar{R}$ as follows:

(7) $$\bar{R}=\frac{2}{\left(N-1\right)N}\sum_{i=1}^{N-1}\sum_{j=i+1}^{N}R_{ij},$$

where N denotes the number of motion waveforms selected to calculate the NCC value. Compared to the NCC value defined in Equation (6), the mean NCC value can comprehensively reflect the waveform similarity by considering all positions of the diaphragm profile.

#### 2.2.4. Motion Limitation Evaluation

**Figure 5:**
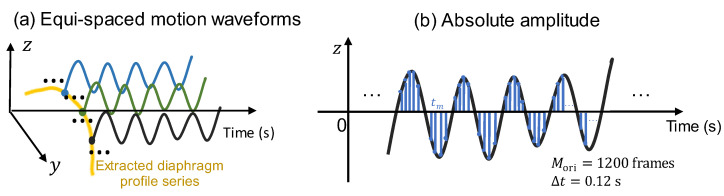
The motion limitation evaluation. (**a**) The equispaced selection of motion waveforms. (**b**) The absolute amplitude calculation by accumulating all the amplitudes in observation time.

In addition to the evaluation approach for motion asynchronization of the diaphragm profile for patients with COPD, an evaluation approach for motion limitation is described in this subsection. Unlike the motion asynchronization evaluation, which requires selecting the waveforms at as many positions of the diaphragm profile on the Y-axis as possible, the limitation evaluation selects the waveforms of partial positions, considering that the waveforms of adjacent positions usually have quite similar amplitude changes. Figure 5a shows the equispaced selection of the motion waveform. More specifically, in the proposed motion limitation evaluation approach, the middle position, $y_{mid}$, for all diaphragm profile series is first determined by:

(8) $$y_{mid}=\left\lfloor \frac{1}{M_{ori}}\sum_{m=1}^{M_{ori}}\left(\frac{1}{N_{ori}(t_{m})}\sum_{i}{f(y_{i},t_{m})y}_{i}\right)+0.5\right\rfloor ,$$

where $1/N_{ori}(t_{m})\sum_{i}{f(y_{i},t_{m})y}_{i}$ denotes the mean Y-axis position of the extracted diaphragm at the time $t_{m}$, and $\left\lfloor \cdot \right\rfloor$ denotes rounding down to an integer. Note that the original diaphragm length, $N_{ori}$, at the m-th moment, and the number of original frames are directly used because pixels of the diaphragm profile always exist at or close to $y_{mid}$ based on our dataset.

For the motion waveform corresponding to $y_{mid}$, we defined the temporal and spatial mean absolute amplitude, which is the observation time, as

(9) $${\bar{z}}_{abs,mean}=\frac{1}{2K+1}\sum_{k=-K}^{K}\left(\frac{1}{M_{ori}}\sum_{m=1}^{M_{ori}}\left|\hat{z}(y_{mid}+{ky}_{int},t_{m})\right|\right).$$

Here, K equispaced motion waveforms on both sides of the middle position, $y_{mid}$, are introduced, and K=5 is set in our approach. Based on the position interval of the Y-axis, $y_{int}=5$ pixels, the final mean absolute amplitude is calculated covering the 11=2K+1 waveforms positioning at $\left[y_{mid}-5y_{int},y_{mid}-4y_{int},\cdots ,y_{mid}+5y_{int}\right]$.

Based on Equation (9), the sampled areas shown in Figure 5b were temporarily meant for each waveform. The accumulated means of all 11 equispaced waveforms were then averaged as the final mean absolute amplitude.

## 3. Experimental Results

### 3.1. Characteristics of Enrolled Participants

The characteristics of 10 normal subjects and 18 COPD patients are shown in Table 2. The COPD patients consisted of 5 GOLD-III and 13 GOLD-IV grades, and the patients were significantly older than the normal subjects. All patients had a smoking history and were significantly different from the non-smoking normal subjects. In PFT, all the test results of COPD patients were significantly lower than those of normal subjects, except for the FRC% predicted.

### 3.2. Verification of Respiration Cycle Differences

**Figure 6:**
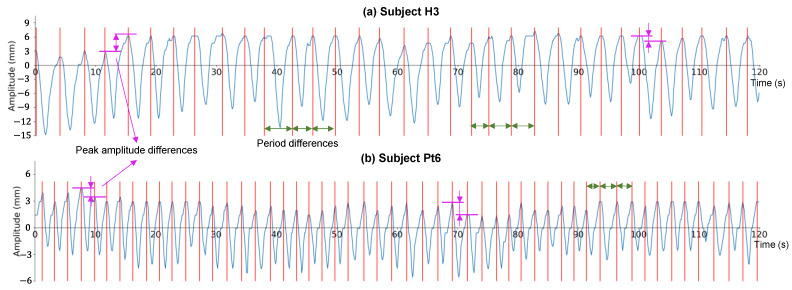
Two examples of motion waveforms at the midpoint of the diaphragm profile. (**a**) A normal subject named H3. (**b**) A chronic obstructive pulmonary disease (COPD) patient named Pt6. Note that the baseline is set as an amplitude of 0.

As examples, the two motion waveforms at the midpoint of the diaphragm profile and the difference in the respiration cycle are shown in Figure 6. The two cases of a normal subject and a patient with COPD are represented by H3 in Figure 6a and Pt6 in Figure 6b, respectively. For the period of the respiration cycle, obvious differences are highlighted at approximately 35–50 s and 70–85 s in Figure 6a and approximately 90–100 s in Figure 6b, as indicated by the green arrows. For the peak amplitude, obvious differences are highlighted at approximately 10–20 s and 95–105 s in Figure 6a, and at approximately 5–15 and 65–75 s in Figure 6b, by the pink arrows. Based on our findings, differences in the period and peak amplitude may appear in both normal subjects and patients with COPD.

**Figure 7:**
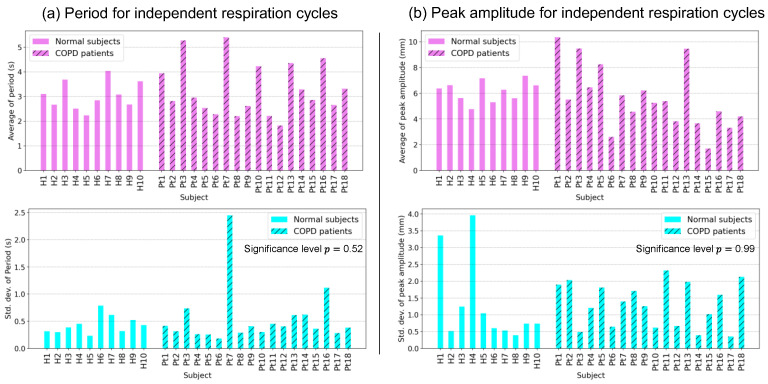
Statistical analysis of independent respiration cycles. (**a**) The averages and standard deviations of the periods of respiration cycles for all subjects. (**b**) The averages and standard deviations of the peak amplitudes of respiration cycles for all subjects.

Figure 7 shows the averages and standard deviations of the period and peak amplitudes of the respiration cycles, as described in Figure 6, for all subjects. Focusing on the standard deviation of each subject, some subjects presented a large value, illuminating the difference between the two parameters of each independent respiration cycle. The averages and standard deviations of the periods of independent respiration cycles are shown in Figure 7a. Large standard deviations were observed for some subjects. Markedly large standard deviations were observed for Pt3, Pt7, and Pt16, suggesting differences in a period in independent respiration cycles. Similarly, the averages and standard deviations of the peak amplitudes of the independent respiration cycles are shown in Figure 7b. Many subjects exhibited large standard deviations. In particular, among all 18 subjects, H1, H4, and Pt2 had the largest standard deviations, which suggested that there were differences in peak amplitude among independent respiration cycles. However, the significance levels of the standard deviation of the period and peak amplitude between normal subjects and COPD patients were 0.52 and 0.99, respectively, which implied that there were no significant differences between the two groups of subjects. A further discussion will be presented in Section 4.1.

### 3.3. Evaluations of Respiratory Diaphragm Motion

The asynchronization and limitation of diaphragmatic motion were evaluated using the mean normalized correlation coefficient and the mean absolute amplitude, respectively.

#### 3.3.1. Motion Asynchronization Evaluation

**Figure 8:**
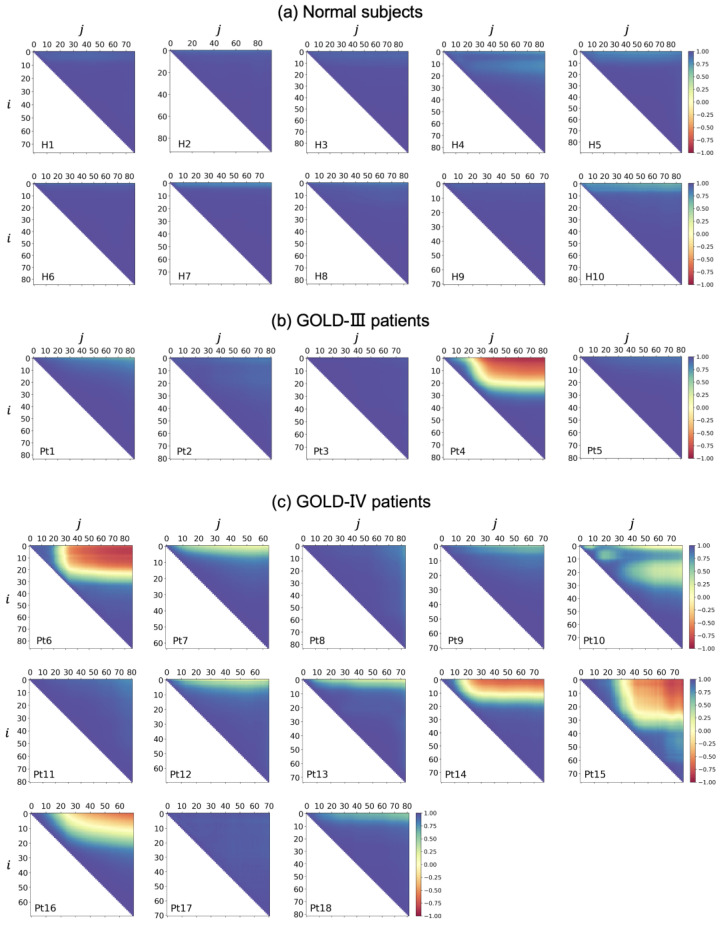
Normalized correlation coefficient (NCC) maps for all subjects. (**a**) Normal subjects. (**b**) COPD Chronic Obstructive Lung Disease (GOLD-III) patients. (**c**) COPD GOLD-IV patients.

Figure 8 shows the NCC maps of all subjects, including normal subjects and patients with COPD. For the ten normal subjects shown in Figure 8a, all of the NCC maps had large NCC values at the half-map at the top right, suggesting motion similarity between the two arbitrary positions of the diaphragm profile. In other words, a synchronized motion was observed for any position of the diaphragm profile in normal subjects. Most of the NCC maps for the five COPD patients with a GOLD-III classification shown in Figure 8b, except for the NCC map of Pt4, had large NCC values at the half-map at the top right, with motion similarity. In contrast, the NCC map of Pt4 had many small NCC values with yellow or red near the top-right corner, suggesting obvious motion asynchronization between the two positions of the diaphragm profile at a certain distance. For the thirteen COPD patients classified as GOLD-IV shown in Figure 8c, we found some relatively small NCC values near the top-right corner of the NCC maps of Pt7 and Pt10, indicated in yellow, suggesting motion asynchronization. Furthermore, small NCC values were found in the NCC maps of Pt6 and Pt14–16, indicated with yellow or red, suggesting obvious motion asynchronization.

**Figure 9:**
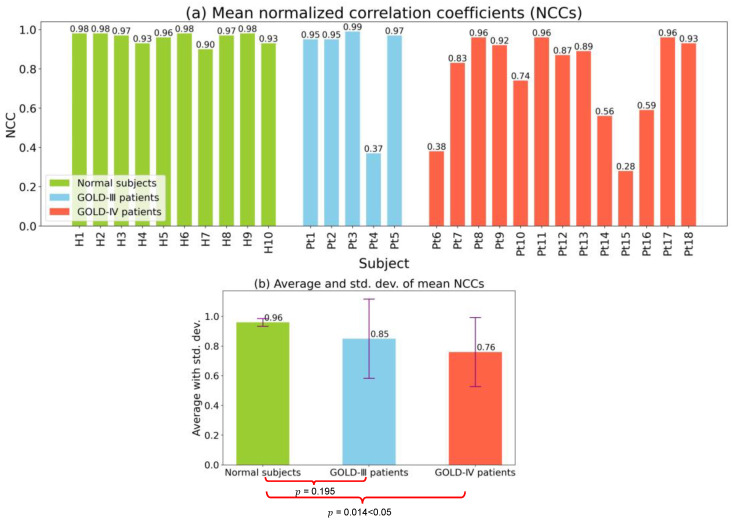
The mean NCC values of diaphragm motion waveforms, and their further averages and standard deviations. (**a**) Mean NCC values for each subject. (**b**) Average and standard deviation of mean NCCs for each group.

The mean NCC values of the diaphragmatic motion waveforms for all subjects and their averages and standard deviations are shown in Figure 9. As shown in Figure 9a, all mean NCC values for normal subjects were quite large, being greater than 0.90. Although most mean NCC values were high for COPD patients with a GOLD-III classification, a small mean NCC of 0.37 was observed for Subject Pt4. Specifically, some small and quite small NCCs existed for the COPD patients with a GOLD-IV classification, especially for Pt6 and Pt15, who had the smallest mean NCC values of 0.38 and 0.28, respectively.

The average and standard deviation of the mean NCC for the three groups of normal subjects, GOLD-III patients, and GOLD-IV patients are shown in Figure 9b. Compared with the mean NCC values of normal subjects, namely 0.96, those of GOLD-III and GOLD-IV patients were smaller at 0.85 and 0.76, respectively. In addition, the standard deviations of the mean NCC values of patients in the GOLD-III and GOLD-IV groups were much larger than those of normal subjects. The averages and standard deviations of mean NCCs also indicated that many small mean NCCs were obtained for GOLD-III and GOLD-IV patients, suggesting asynchronization motions of the diaphragm. Specifically, we conducted a significance test of the mean NCC values between normal subjects and GOLD-III or GOLD-IV patients and obtained p-values of 0.195 and 0.014, respectively. A p-value less than 0.05 implied that there was a difference in the mean NCC values between normal subjects and GOLD-IV patients.

#### 3.3.2. Motion Limitation Evaluation

**Figure 10:**
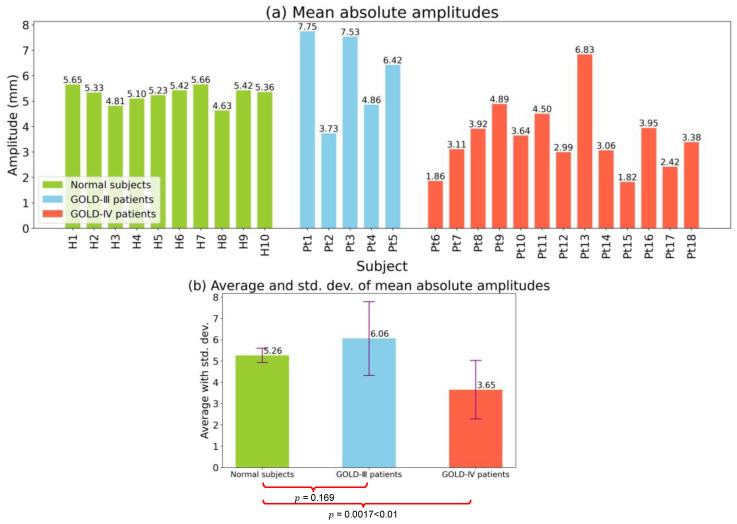
The mean absolute amplitudes of diaphragm motion waveforms, and their further averages and standard deviations. (**a**) Mean absolute amplitudes for each subject. (**b**) Average and standard deviation of mean absolute amplitudes for each group.

Similar to Figure 9, the mean absolute amplitudes and their averages and standard deviations of the diaphragm motion waveforms are shown in Figure 10. As shown in Figure 10a, the mean absolute amplitudes of all ten normal subjects were distributed within a concentrated range of 4.63–5.56. In contrast, there were obvious differences in the mean absolute amplitudes among COPD patients with a GOLD-III classification. Specifically, Pt2 had a relatively small mean absolute amplitude of only 3.73. Furthermore, among COPD patients with a GOLD-IV classification, smaller mean absolute amplitudes were observed. Specifically, the smallest amplitudes, observed for patients Pt6 and Pt15, were smaller than 2.00, i.e., 1.86 and 1.82, respectively.

The averages and standard deviations of the mean absolute amplitudes for the three groups of normal subjects, GOLD-III patients, and GOLD-IV patients are depicted in Figure 10b. GOLD-IV patients showed a mean absolute amplitude of only 3.65, which was much smaller than the mean absolute amplitudes for normal subjects and GOLD-III patients, which were 5.26 and 6.06, respectively. Moreover, GOLD-III and GOLD-IV patients had larger standard deviations than normal subjects. Based on the averages and standard deviations of the mean absolute amplitude, it was clear that there were many small amplitudes for GOLD-III and GOLD-IV patients, suggesting limited motion of the diaphragm. Specifically, a significant difference test was conducted on the mean absolute amplitudes between normal subjects and GOLD-III or GOLD-IV patients, and the p-values were calculated as 0.169 and 0.0017, respectively. A significant difference in the mean absolute amplitude between normal subjects and GOLD-IV patients was demonstrated by a p-value of 0. 0017, which is less than 0.01. Our findings regarding low-motion amplitudes of the diaphragm are consistent with those of previous studies [32,33].

#### 3.3.3. Multivariate Analysis

**Figure 11:**
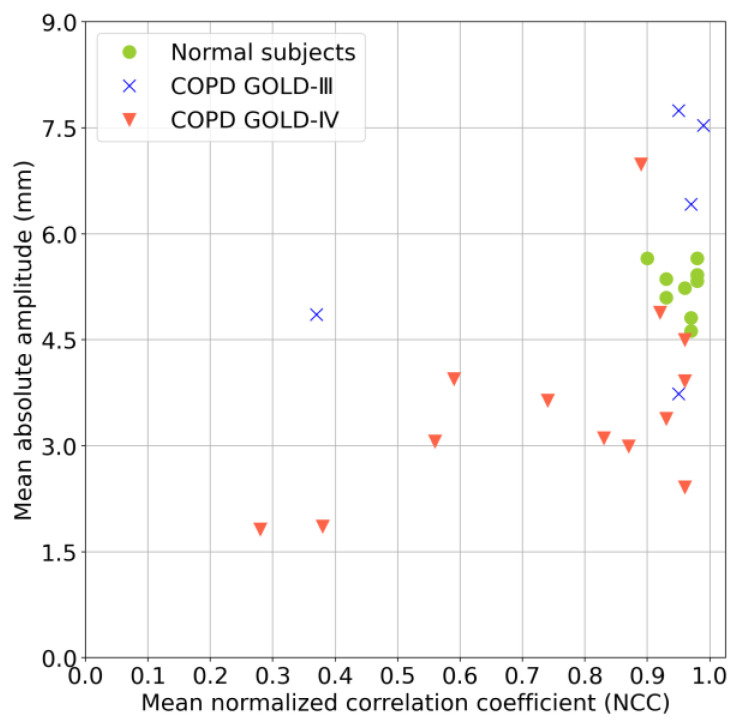
Multivariate analysis of mean NCC values and mean absolute amplitudes.

The results of a multivariate analysis combining the mean NCC values and mean absolute amplitudes are shown in Figure 11, referring to some related multivariate analyses, such as those in [29,34]. This analysis provided a more comprehensive comparison of the diaphragm motions of normal subjects and patients with COPD. The mean NCC values and the mean absolute amplitudes for normal subjects were within the ranges of 0.9–1.0 and 4.5–6.0, respectively. In contrast, samples for the COPD patients with a GOLD-III classification were dispersed, with a mean NCC value of less than 0.4 and a mean absolute amplitude of less than 4.5. Specifically, the samples of COPD patients with a GOLD-IV classification were more dispersed, with mean NCC values and mean absolute amplitudes of most samples less than 0.9 and 4.5, respectively.

## 4. Discussion

### 4.1. Verification of Respiration Cycle Differences

After reviewing the standard deviations of the periods and peak amplitudes of all independent respiration cycles in each subject, with p-values of 0.52 and 0.99, respectively, between normal subjects and COPD patients, as shown in Figure 7, two findings are obtained. The first is that there are obviously high standard deviations among all subjects; that is, the periods and peak amplitudes of their independent respiration cycles have essential differences, which implies the necessity of motion analysis on multiple cycles. Second, the p-values for comparisons between normal subjects and patients with COPD indicated no significant difference, which implies that the variation between each respiration cycle within an individual subject is not unique to COPD. Note that, unlike the peak amplitude, COPD may bring about differences in the absolute amplitudes between the normal subjects and the COPD patients, for the motion limitation evaluation of the diaphragm.

### 4.2. Regional Diaphragm Asynchronization Motion Corresponding to the NCC Map

**Figure 12:**
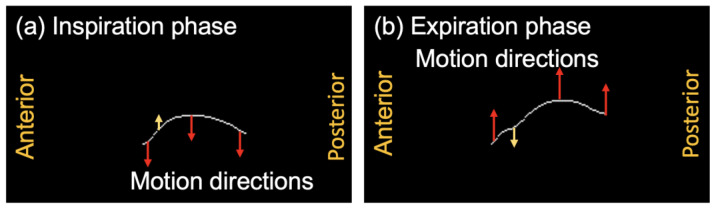
The motion directions of the extracted diaphragm profiles of Subject Pt10 belonging to the COPD GOLD-IV patient group. Yellow arrows present the opposite motion directions with red arrows. (**a**) Inspiration phase. (**b**) Expiration phase.

Figure 12 illustrates the motion directions of each position in the extracted diaphragm profiles of Pt10, who belongs to the COPD GOLD-IV group. In the inspiration phase shown in Figure 12a, unlike the downward motion directions of most other positions, the upward motion near the anterior position is in the opposite direction, as highlighted by the yellow arrow. Similarly, in the expiration phase shown in Figure 12b, the downward motion near the anterior position exhibited a direction opposite to the upward motion directions of most other positions. The corresponding diaphragmatic asynchronization motion of two respiratory phases between the anterior and other positions is shown in Figure 12. The asynchronized motion is also verified by the NCC map of Pt10 in Figure 8c, in which low NCC values were observed at the positions of the small range of index i, and the large range of index j.

### 4.3. Limitation

This study has several limitations. First, during the pre-processing of the diaphragm profile extraction, the image texture information of the lung and liver around the diaphragm is neglected, which may assist in the motion evaluation of the diaphragm. In this study, although we evaluated the vertical motions of each position of the diaphragm, which are typically the most dominant, motion estimations in other directions were not obtained because they were limited by the extracted diaphragm profiles. The importance and estimation method of the multiple-direction-based diaphragm evaluation should be further explored in future studies. Second, our data do not come from a huge number of subjects, and almost all the patients had severe to very severe COPD. The result should be confirmed in a large sample size that targets all stages of COPD.

## 5. Conclusions and Future Work

In this study, a novel method for evaluating respiratory diaphragm motion is proposed to assess the asynchronization and limitation motions of patients with COPD. Compared to most conventional lung or diaphragm motion evaluation methods, which usually deal with very few respiration cycles, our proposed method assesses multiple respiration cycles, resulting in a more robust evaluation through a comprehensive assessment. Using the motion waveforms generated from the extracted diaphragm profile series, the differences in the respiration cycle were verified, and the motion asynchronization and limitations of patients were evaluated using the NCC value and absolute amplitude, respectively. The experimental results demonstrated the abnormal diaphragm motions of COPD patients compared with normal subjects, which would assist in COPD diagnosis and feedback regarding COPD therapy and patient rehabilitation.

Compared with the COPD patients in GOLD-III, the more severe patients in GOLD-IV were verified to have more obvious motion limitations. In the future, we will explore the mechanism and relationship between the limitation of diaphragm motion and the extent of airway obstruction [35].

## Figures and Tables

**Table 1:** Parameter settings for MRI system.

Parameter	Specification
Repetition time	1.84 mms
Echo time	0.71 MS
Flip angle	45°
Image size	256 × 256 pixel
Field of view	384 × 384 mm
Resolution	1.5 mm/pixel
Slice thickness	13.5 mm
Frame rate	8.33 f/s
Measurement time for each subject	More than 2 min
No. of frames for each subject	1200

**Table 2:** Characteristics of all the enrolled participants.

Parameter	Normal (n = 10)	COPD (n = 18)	*p*-Value
Age (year)	31.8±1.5	67.4±9.3	<0.0001 *
Male sex (%)	10 (100)	16 (89)	
BMI (kg/m^2^)	21.5±1.2	19.4±3.1	0.06
Pack-years	0	61±32	<0.0001 *
GOLD grade (I/II/III/IV)	N. A	0 (0%)/0 (0%)/5 (27.8%)/13 (72.2%)	
**Pulmonary Function Tests (PFTs)**			
FVC% predicted (%)	102.2±14.5	73.5±18.8	0.0006 *
FEV_1_ (L)	4.23±0.4	0.83±0.28	<0.0001 *
FEV_1_% predicted (%)	99.9±11.5	30.8±9.3	<0.0001 *
FEV_1_/FVC (%)	87.0±6.3	34.0±5.9	<0.0001 *
FRC% predicted (%)	120.9±15.2	131.0±18.8	0.2
RV% predicted (%)	123.9±38.0	159.0±29.4	0.02 *
RV/TLC (%)	27.9±7.8	53.5±8.4	<0.0001 *
YHXSYXDLco/VA% predicted (%)	114.9±11.4	52.1±22.4	<0.0001 *

* p<0.05. Data are expressed as means ± standard deviation. The smoking status is shown in pack-years calculated by multiplying the number of packs of cigarettes the person smoked per day by the number of years the one has smoked. Abbreviations—COPD: chronic obstructive pulmonary disease; BMI: body mass index; GOLD: global initiative for chronic obstructive lung disease; FVC: forced vital capacity; FEV1: forced expiratory volume in 1 s; FRC: functional residual capacity; RV: residual volume; TLC: total lung capacity; DLco/VA: diffusing capacity for carbon monoxide per liter.

## Data Availability

To avoid any potential breach of patient confidentiality, the data were deidentified and had no linkage to the researchers.
